# Purification, Identification, and *In Silico* Analysis of Anti-Obesity and Antidiabetic Peptides from the Red Seaweed *Palmaria palmata*

**DOI:** 10.3390/md23100392

**Published:** 2025-10-03

**Authors:** Sakhi Ghelichi, Mona Hajfathalian, Seyed Hossein Helalat, Birte Svensson, Charlotte Jacobsen

**Affiliations:** 1 Research Group for Bioactives–Analysis and Application, National Food Institute, Technical University of Denmark, 2800 Kongens Lyngby, Denmark; monhaj@food.dtu.dk; 2 Department of Health Technology, Technical University of Denmark, 2800 Kongens Lyngby, Denmark; helalat@dtu.dk; 3 Enzyme and Protein Chemistry, Department of Biotechnology and Biomedicine, Technical University of Denmark, 2800 Kongens Lyngby, Denmark; bis@bio.dtu.dk

**Keywords:** *Palmaria palmata*, bioactive peptides, lipase, α-amylase, enzymatic/alkaline extraction, *in silico* analysis

## Abstract

This study investigates the anti-obesity and antidiabetic potential of *P. palmata* extracts produced through sequential enzymatic and alkaline treatments. Among the treatment groups, the extract treated solely with Alcalase^®^ (Alc) demonstrated the highest protein content (10.11 ± 0.15%) and degree of hydrolysis (30.36 ± 0.77%), significantly outperforming other treatments (*p* < 0.05). The Alc extract also exhibited superior inhibitory activity against porcine pancreatic lipase and α-amylase, achieving the lowest IC_50_ for lipase (2.29 ± 0.87 mg.mL^−1^) and showing significant enzyme inhibition across all tested concentrations (*p* < 0.05). Ultrafiltration of the Alc extract revealed that peptide fractions < 1 kDa and 1–3 kDa were most effective in enzyme inhibition, with IC_50_ values of 3.25–3.55 mg.mL^−1^ for both lipase and α-amylase. Peptides were identified via LC-MS/MS analysis and database searching using SequestHT, resulting in 536 sequences, of which bioinformatic screening yielded 51 non-toxic, non-allergenic candidates (PeptideRanker score > 0.6); four of these contained known inhibitory motifs for lipase and α-amylase. Molecular docking confirmed strong binding affinities between these peptides and their respective enzymes, supporting their potential as natural enzyme inhibitors. These findings indicate the functional food potential of Alcalase^®^-derived *P. palmata* peptides for managing obesity and type 2 diabetes.

## 1. Introduction

In recent decades, the global surge in obesity and diabetes has emerged as a major public health crisis, placing significant strain on healthcare systems and being strongly associated with a wide range of medical conditions, especially cardiovascular diseases [1]. Due to the challenges involved in managing these severe problems and the risks associated with conventional drug therapies and surgical procedures, interest has increasingly shifted toward natural substances that may offer anti-obesity and antidiabetic effects [2,3]. Among the various natural compounds under investigation, bioactive peptides have gained considerable attention as potential therapeutic agents [4]. These peptides are typically released through the enzymatic hydrolysis of proteins derived from food sources such as dairy, marine organisms, eggs, and plants, and are being extensively studied for their ability to modulate key metabolic pathways involved in the management of obesity and diabetes [5,6]. 

The inhibition of key metabolic enzymes plays a pivotal role in the search for anti-obesity and antidiabetic peptides [7]. Targeting enzymes involved in lipid and carbohydrate metabolism, such as pancreatic lipase, which facilitates fat digestion, and α-amylase, which breaks down dietary carbohydrates (specifically starch and dextrins), has emerged as a promising strategy [8]. Bioactive peptides capable of inhibiting these enzymes offer a potential therapeutic approach for managing obesity and preventing the onset of diabetes [1]. Notably, such bioactive peptides derived from marine organisms have demonstrated potential anti-obesity and antidiabetic properties through their inhibitory effects on key metabolic enzymes. Inhibition of porcine pancreatic lipase and α-amylase has been reported for peptides derived from fish, oyster, and sea cucumber [9,10,11,12,13,14].

Among aquatic sources, seaweed is considered one of the most promising to produce bioactive peptides. Studies have shown that seaweed-derived peptides can inhibit lipase [15] and α-amylase [16,17], indicating their potential role in obesity and diabetes intervention strategies. Red seaweed, particularly due to its relatively high protein content, represents a promising source of bioactive peptides [18]. Among red seaweeds, *Palmaria palmata*, commonly known as dulse, is of particular interest. This species is included in the list of macroalgae authorized for food use in the European Union under Regulation (EU) 2017/2470 and benefits from regulatory acceptance, which enhances its potential for functional food applications [19]. Extracts from *P. palmata* have been reported to exhibit inhibitory activity against metabolic enzymes such as lipase and α-amylase, indicating their potential as a source of anti-obesity and antidiabetic peptides [15,20,21]. However, to date, no studies have isolated and identified the specific peptides responsible for this activity. Therefore, the aim of this study is to evaluate various enzymatic/alkaline extraction treatments to determine the optimal conditions for obtaining extracts with the highest lipase and α-amylase inhibitory activities. The treatments applied included: no enzyme (No Enz), Alcalase^®^ (Alc; a broad-specificity serine endopeptidase), Formea^®^ Prime (Form; a protease with trypsin-like specificity), and a combination of Alcalase^®^ and Formea^®^ Prime (Alc+Form), each followed by alkaline extraction. The most active extract was subsequently subjected to peptide purification and sequence identification. Furthermore, the bioactive properties of the identified peptides were investigated through *in silico* analyses and molecular docking studies.

## 2. Results 

### 2.1. Protein Content and Degree of Hydrolysis (DH)

**Figure 1:**
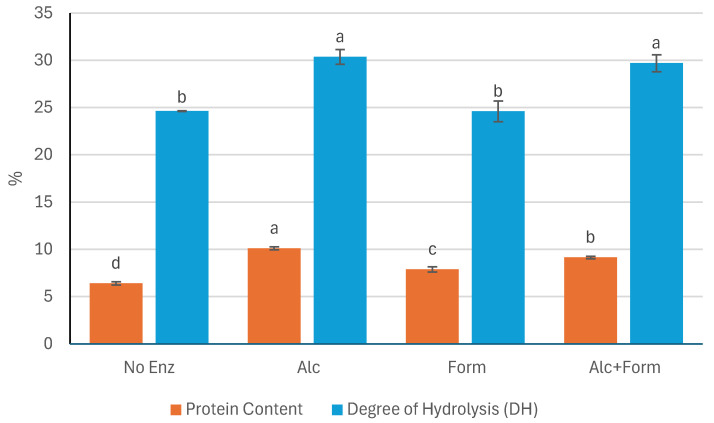
Protein content (%) and degree of hydrolysis (DH, %) of *P. palmata* extracts after sequential enzymatic and alkaline treatments using different enzymatic treatments. No Enz, Alc, Form, and Alc+Form refer to treatments with no enzyme, Alcalase^®^, Formea^®^ Prime, and a combination of Alcalase^®^ and Formea^®^ Prime, respectively. Data were expressed as mean ± standard deviation (*n* = 3 for protein content and *n* = 2 for DH). Different letters denote significant differences among the treatments in terms of protein content and DH (*p* < 0.05).

The protein content (expressed as % dry matter) and the DH (%) of *P. palmata* extracts following sequential enzymatic and alkaline treatments are presented in Figure 1. Significant differences in protein content were observed among the treatments, following the order: Alc > Alc+Form > Form > No Enz (*p* < 0.05). The extract treated with Alcalase^®^ alone (Alc) yielded the highest protein content (10.11 ± 0.15%), indicating a more efficient release of soluble proteins under this condition.

Similarly, the highest DH values were obtained in the samples treated with Alcalase^®^ (Alc with 30.36 ± 0.77% and Alc+Form with 29.70 ± 0.89%), both of which showed significantly greater hydrolysis compared to Form (24.60 ± 1.09%) and No Enz (24.63 ± 0.04%) (*p* < 0.05). However, no significant difference in DH was observed between the Alc and Alc+Form treatments (*p* > 0.05), suggesting that the addition of Formea^®^ Prime did not enhance proteolysis beyond what was achieved with Alcalase^®^ alone. In contrast, the Form and No Enz treatments exhibited the lowest DH values, with no significant difference between them (*p* > 0.05), indicating minimal protein hydrolysis in the absence of Alcalase^®^.

### 2.2. Anti-Obesity and Antidiabetic Properties of Extracts

The anti-obesity properties of the enzymatic/alkaline extracts of *P. palmata*, obtained through different enzymatic treatments, are presented in Table 1. These properties were assessed based on the extracts’ ability to inhibit the metabolic enzyme porcine pancreatic lipase across a range of concentrations (4.0 to 0.125 mg.mL^−1^). At all concentrations tested, the extract treated with Alcalase^®^ alone (Alc) exhibited significantly higher lipase inhibitory activity compared to the other treatments (*p* < 0.05), except for the Alc+Form treatment at 4.0 mg.mL^−1^, where no significant difference was observed (*p* > 0.05). Among all treatments, only Alc achieved at least 50% inhibition of lipase activity at the highest concentration tested (4.0 mg.mL^−1^), with an IC_50_ value of 2.29 ± 0.87 mg.mL^−1^. In contrast, the other extracts did not reach the 50% inhibition threshold within the tested concentration range, indicating limited lipase inhibitory potential under the given conditions. The application of Alcalase^®^ clearly enhanced the lipase inhibitory activity of the extracts compared to treatments where Alcalase^®^ was not used (*p* < 0.05). Interestingly, the combination of Alcalase^®^ with Formea^®^ Prime (Alc+Form) resulted in significantly lower inhibitory activity than Alcalase^®^ alone (*p* < 0.05), suggesting a possible interaction or dilution effect that reduced efficacy. Furthermore, no significant differences were observed between the Form and No Enz treatments across all tested concentrations (*p* > 0.05), except at 0.5 mg.mL^−1^, where Form demonstrated significantly lower lipase inhibition than even the No Enz control (*p* < 0.05). 

The antidiabetic potential of *P. palmata* enzymatic/alkaline extracts, produced using various enzymatic treatments, is summarized in Table 2. This activity was evaluated through the extracts’ capacity to inhibit porcine pancreatic α-amylase over a concentration range of 4.0 to 1.0 mg.mL^−1^. Concentrations below 1.0 mg.mL^−1^ were not tested, as preliminary observations indicated minimal inhibitory effects at that level, rendering lower concentrations scientifically irrelevant for further analysis. Among all treatments, the extract prepared with Alcalase^®^ alone (Alc) consistently demonstrated the highest α-amylase inhibition across the tested concentrations (*p* < 0.05). An exception was at 1.0 mg.mL^−1^, where Alc did not show a statistically significant difference compared to the No Enz control (*p* > 0.05). Notably, none of the extracts achieved 50% inhibition of α-amylase at the maximum concentration tested (4.0 mg.mL^−1^), and therefore, IC_50_ values could not be determined under these conditions. As observed in the lipase inhibition results, the use of Alcalase^®^ significantly improved the antidiabetic activity of the extracts compared to treatments lacking this enzyme (*p* < 0.05). However, combining Alcalase^®^ with Formea^®^ Prime (Alc+Form) did not enhance inhibitory activity and, in fact, produced results statistically comparable to the No Enz treatment (*p* > 0.05), except at 1.0 mg.mL^−1^, where the inhibitory activity of Alc+Form was significantly lower than that of No Enz (*p* < 0.05). Furthermore, the Form-treated extract consistently exhibited the lowest α-amylase inhibition among all treatments, with significantly reduced activity compared to the others across all concentrations tested (*p* < 0.05).

### 2.3. Anti-Obesity and Antidiabetic Properties of Fractions Obtained from Enzymatic/Alkaline Extracts Using Alcalase^®^

Based on the inhibitory activities against lipase and α-amylase of the extracts obtained through sequential enzymatic and alkaline treatments, the extract produced using only Alcalase^®^ was selected for further analysis due to its superior inhibitory effects relative to other treatments. The inhibitory activity and corresponding IC_50_ values of the resulting peptide fractions obtained by ultrafiltration of the selected extract are presented in Table 3. The molecular weight ranges of the fractions were <1 kDa, 1–3 kDa, 3–5 kDa, and >5 kDa. The highest inhibition of porcine pancreatic lipase was observed in the <1 kDa and 1–3 kDa fractions across all tested concentrations, with significantly greater activity than the 3–5 kDa and >5 kDa fractions (*p* < 0.05). The IC_50_ values for lipase inhibition were 3.25 mg.mL^−1^ and 3.47 mg.mL^−1^ for the <1 kDa and 1–3 kDa fractions, respectively (*p* < 0.05). In contrast, the IC_50_ values for the 3–5 kDa and >5 kDa fractions could not be determined, as they did not reach 50% inhibition at the highest tested concentration (4 mg.mL^−1^). These findings indicate that lipase inhibitory activity declines with increasing peptide size.

A similar molecular weight-dependent trend was observed in α-amylase inhibition. The <1 kDa and 1–3 kDa fractions exhibited significantly higher inhibitory activity compared to the 3–5 kDa and >5 kDa fractions at all tested concentrations (*p* < 0.05). The IC_50_ values for α-amylase inhibition were 3.24 mg.mL^−1^ and 3.55 mg.mL^−1^ for the <1 kDa and 1–3 kDa fractions, respectively (*p* < 0.05), while IC_50_ values were not reached for the higher molecular weight fractions at 4 mg.mL^−1^. Interestingly, while the 3–5 kDa and >5 kDa fractions demonstrated moderate lipase inhibition at the highest concentration tested (46.27% and 41.33%, respectively), their α-amylase inhibitory activities were substantially lower at the same concentration (29.68% for the 3–5 kDa fraction and only 6.78% for the >5 kDa fraction). In contrast, the <1 kDa and 1–3 kDa fractions showed significantly higher α-amylase inhibition at 58.25% and 55.11%, respectively at 4 mg.mL^−1^ (*p* < 0.05).

### 2.4. In Silico Prediction and Docking Analysis of Peptides

#### 2.4.1. Bioactivity Prediction

All identified peptides were subjected to bioactivity screening using the PeptideRanker tool. Of the 536 peptides analyzed, 51 exhibited high bioactivity scores (>0.6), indicating strong potential for biological activity. The complete list of these 51 peptides, along with their predicted bioactivity scores and parent protein accession numbers, is provided in Supplementary Material Table S1.

#### 2.4.2. Toxicity and Allergenicity Assessment

The 51 shortlisted peptides were further evaluated for potential toxicity using ToxinPred. All peptides were predicted to be non-toxic, suggesting a favorable safety profile for application in functional foods. Allergenicity prediction was performed using AllerCatPro 2.0 and AllergyPred. Peptides classified as non-allergenic by both tools were retained for subsequent analysis, while any peptide flagged by either tool was excluded. The final peptide set consisted exclusively of non-toxic, non-allergenic sequences.

#### 2.4.3. Functional Activity Screening

**Figure 2:**
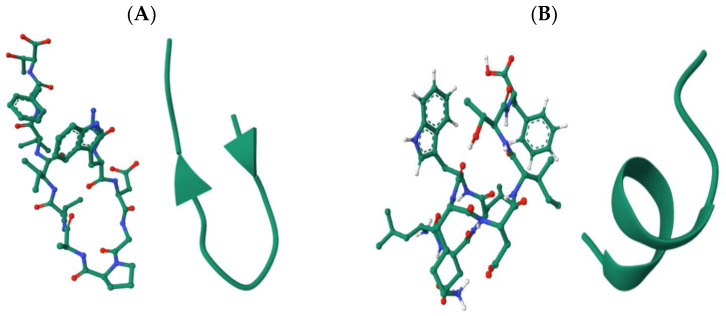

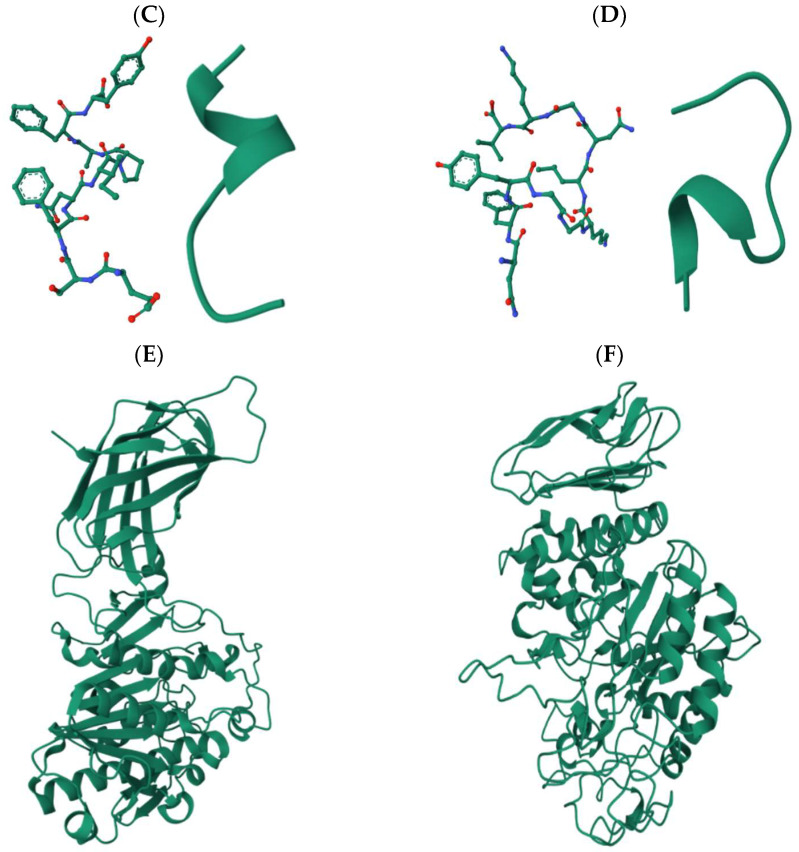
Cartoon and ball-and-stick 3D models of the selected peptide sequences: (**A**) SWDGPALVVFT and (**B**) LDLWKDITF, targeting pancreatic lipase inhibition; (**C**) ESFNIPAFY and (**D**) NFYGGKLNGKV, targeting pancreatic α-amylase inhibition; (**E**) Structure of human pancreatic lipase (PDB ID: 2PPL); and (**F**) Structure of human pancreatic α-amylase (PDB ID: 1HNY).

The validated peptides were screened for known bioactive motifs using the BIOPEP-UWM database. This analysis identified two peptides, SWDGPALVVFT and LDLWKDITF, with potential lipase inhibitory activity, and two peptides, ESFNIPAFY and NFYGGKLNGKV, with predicted pancreatic α-amylase inhibitory activity. Although NFYGGKLNGKV displayed a PeptideRanker score below the bioactivity threshold of 0.6, it was retained for further analysis due to its predicted α-amylase inhibitory potential based on motif analysis. To gain structural insight into these bioactive peptides, three-dimensional (3D) models were generated, and their spatial conformations are illustrated in Figure 2. Peptides predicted to inhibit lipase (SWDGPALVVFT and LDLWKDITF) are presented in panels A and B, while those targeting α-amylase inhibition (ESFNIPAFY and NFYGGKLNGKV) are shown in panels C and D, respectively. Panels E and F show the crystal structures of human pancreatic lipase (PDB ID: 2PPL) and human pancreatic α-amylase (PDB ID: 1HNY), respectively, to contextualize the docking and interaction analyses performed in later sections.

In addition to structural modeling, key physicochemical properties of the selected peptides were analyzed, including molecular weight, isoelectric point, net charge at physiological pH, and estimated solubility. These properties are summarized in Table 4, offering a preliminary assessment of each peptide’s biochemical behavior and potential for bioavailability.

#### 2.4.4. Molecular Docking Analysis

Molecular docking studies were carried out using ClusPro and SwissDock to investigate the interaction of the selected peptides with their target enzymes. The peptides SWDGPALVVFT and LDLWKDITF were docked to human pancreatic lipase (PDB ID: 2PPL), yielding AC/SwissParam scores of 180.4/−9.1 and 77.7/−7.5, respectively. These scores represent the binding affinity and interaction energy between the peptides and the enzyme, where a higher AC score and a more negative SwissParam score indicate stronger and more favorable binding interactions, suggesting potential inhibitory activity against the lipase. The peptides were found to occupy a similar binding cavity within the enzyme’s active site, with consistent interaction patterns suggesting comparable modes of inhibition. Furthermore, the peptides ESFNIPAFY and NFYGGKLNGKV were docked to human pancreatic α-amylase (PDB ID: 1HNY). The estimated AC/SwissParam binding scores were 1068.3/51.9 and 3082.5/135, respectively. Both peptides formed multiple stable hydrogen bonds and hydrophobic interactions, indicating strong binding affinity and potential inhibitory activity.

Docking positions the peptide SWDGPALVVFT across the lid/hinge surface that controls interfacial activation. The Trp2 in the peptide and Pro41 in the enzyme anchors the peptide at the mouth of the cleft, while the C-terminal Thr11-Asp267 interaction pins the hinge through a directional H-bond. The hydrophobic core (Val8-Val264 and Phe10-Leu266) packs along the nonpolar face of the lid, stabilizing a closed/less-open conformation that occludes substrate access rather than engaging the Ser-His-Asp catalytic triad directly. This topology predicts Vmax suppression (non-competitive or mixed inhibition). The peptide LDLWKDITF adopts a bridging pose that links the rim of the catalytic cleft to the lid/hinge interface. At the cleft rim, Leu1-Glu197 and Leu3-Ile228 provide a hydrophobic/polar anchor, while the aromatic Trp4 establishes multiple contacts to the entry surface (Ile39/Arg40/Pro41) and Thr134, consistent with π-cation and CH-π stabilization at the pocket mouth. The C-terminal Phe9 reaches the hinge (Asp267 and Thr134), complementing the Trp node to form a two-point latch that restrains lid opening and sterically impedes substrate ingress. As with SWDGPALVVFT, the pattern of contacts argues for interfacial (lid-locking) inhibition with a primary effect on Vmax, preference for the closed state. Taken together, both peptides behave as lid-locking inhibitors that stabilize non-productive conformations rather than competing at the catalytic Ser-His-Asp triad.

**Figure 3:**
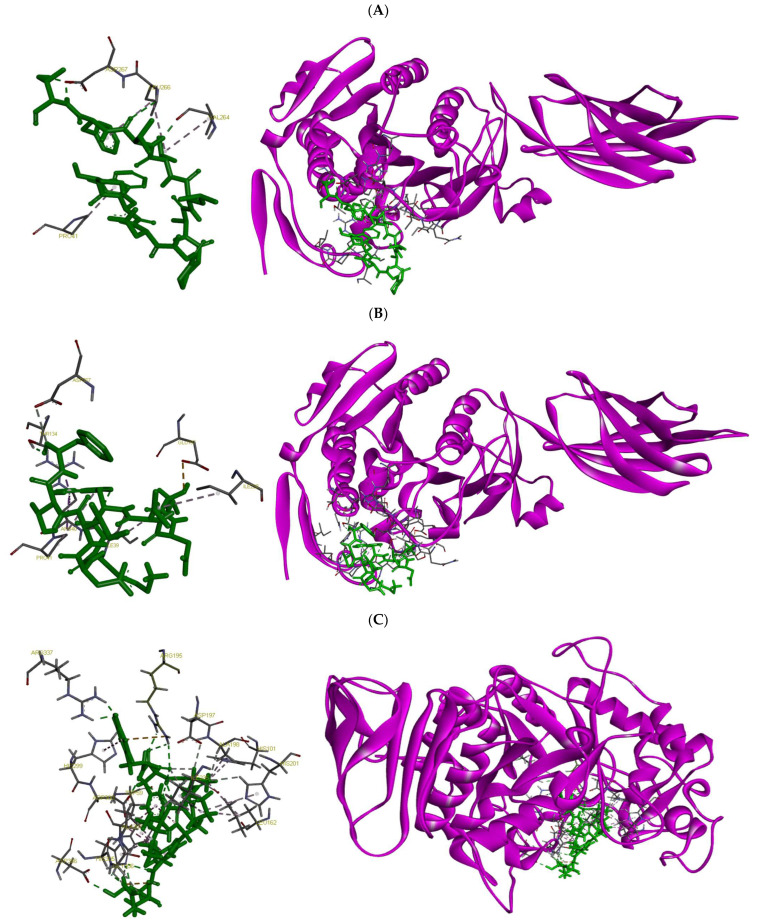

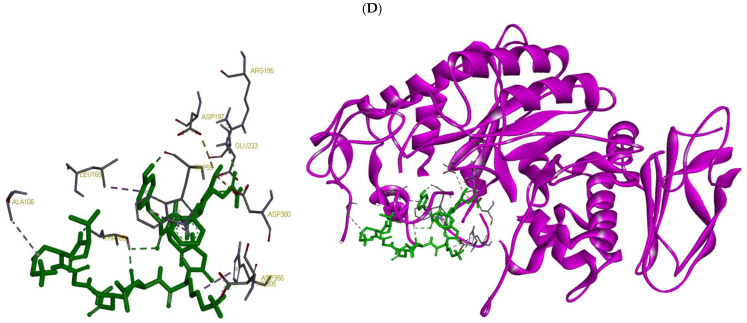
Molecular docking results showing the binding interactions of selected peptides with their respective digestive enzyme targets: (**A**) SWDGPALVVFT and (**B**) LDLWKDITF docked onto human pancreatic lipase; and (**C**) ESFNIPAFY and (**D**) NFYGGKLNGKV docked onto human pancreatic α-amylase.

Docking places the peptide ESFNIPAFY in the catalytic cleft spanning the glycone and aglycone subsites, with direct contacts to the catalytic acids Asp197 and Asp300 that are consistent with a competitive, active-site–blocking mechanism. The peptide’s residues anchor against Trp59 and pack near Leu162, stabilizing the pose, while additional polar contacts to His299/His305 align over the catalytic water network. Contacts with Asp356 further extend into the region. Notably, interactions with Arg195 and Arg337, key residues of the chloride-activation pocket, suggest the peptide could perturb Cl^-^ coordination, thereby attenuating catalytic turnover in addition to steric occlusion. A contact near His201 (within the Ca^2+^-stabilized loop) may further rigidify the active-site architecture. Together, the Asp197/Asp300 engagement, Trp59 aromatic stacking, and Arg195/Arg337 contacts explain how ESFNIPAFY is predicted to suppress α-amylase activity by blocking substrate entry and disrupting essential catalytic/cofactor networks. The peptide NFYGGKLNGKV docks deeper across the catalytic triad, Asp197, Glu233, and Asp300 forming an extended hydrogen-bond/ionic network that directly disfavors glycosidic bond cleavage. The peptide provides π-stacking with Trp59, while interactions with Thr163 and Leu165 shape the peptide along the channel—additional contacts with His305 and Asp356 reinforce occupancy of the aglycone subsites. Engagement of Arg195 (chloride pocket) suggests partial interference with the Cl^−^-dependent activation pathway, complementing the orthosteric block at the acids. The presence of two Lys residues offers favorable electrostatic pairing with the catalytic carboxylates, stabilizing the inhibitor pose. Overall, the combination of triad engagement (Asp197/Glu233/Asp300), Trp59 stacking, and subsite contacts supports a competitive inhibition model that both occludes substrate binding and disrupts transition-state stabilization (Figure 3).

## 3. Discussion

### 3.1. Protein Content and Degree of Hydrolysis

The results of this study demonstrate that the application of Alcalase^®^ significantly increased the solubilized protein content in the seaweed extracts. Among all treatments, the extract obtained through sequential enzymatic-alkaline treatment with Alcalase^®^ alone exhibited the highest protein content. This was significantly greater than the extract obtained with the combination of Alcalase^®^ and Formea^®^ Prime, followed by Formea^®^ Prime alone, and finally the treatment without any enzymatic input (*p* < 0.05). These findings align with previous work on *P. palmata*, which showed that the use of Alcalase^®^, either independently or in combination with other enzymes, consistently resulted in higher protein yields [19]. The efficacy of Alcalase^®^ in seaweed protein extraction is likely due to its broad substrate specificity and robust proteolytic activity, which allows it to break down protein structures that are otherwise tightly embedded within the seaweed cell wall matrix. Alcalase^®^, a serine endopeptidase from *Bacillus licheniformis*, is known for its efficiency in hydrolyzing both internal and terminal peptide bonds, making it particularly effective for processing protein-rich biomass such as seaweeds [22]. In contrast, the comparatively lower protein content in extracts treated with Formea^®^ Prime, either alone or in combination with Alcalase^®^, may be attributed to its high specificity, cleaving only at the C-terminal side of lysine or arginine (except when followed by proline), and its activity being influenced by factors such as pH, temperature, protein structure, autolysis, and organic solvents [23]. Seaweed proteins may lack sufficient exposure or frequency of lysine and arginine residues in accessible regions, and the alkaline conditions used during extraction could further limit trypsin’s activity by affecting its stability and efficiency. As a result, trypsin’s effectiveness in disrupting the protein matrix appears to be limited in this context. 

Enzymatic treatment with Alcalase^®^ significantly outperformed Formea^®^ Prime in terms of both DH and protein solubilization. Alcalase^®^’s broad substrate specificity allows it to cleave a wide range of peptide bonds, generating a hydrolysate rich in small peptides and thereby enhancing protein recovery from structurally complex substrates such as red macroalgae. Several studies have supported its use for producing protein hydrolysates with high yields and desirable functional properties from various seaweed biomasses [24,25,26,27]. In contrast, Formea^®^ Prime, a trypsin-like protease with narrower specificity, exhibited lower hydrolytic efficiency, which aligns with previous studies showing trypsin’s limited ability to degrade certain complex proteins [28,29,30]. This difference features the importance of selecting proteases based on substrate complexity to maximize extraction yields.

### 3.2. Anti-Obesity and Antidiabetic Properties of Enzymatic/Alkaline Extracts

The enhanced lipase inhibitory activity observed in extracts treated with Alcalase^®^ highlights the enzyme’s key role in releasing or generating bioactive compounds capable of modulating pancreatic lipase, a crucial enzyme in dietary fat digestion. The broad proteolytic activity of Alcalase^®^ likely generates bioactive peptides with enhanced affinity for pancreatic lipase, aligning with previous reports that associate Alcalase^®^-mediated hydrolysis with anti-obesity properties in protein hydrolysates [29,30,31,32]. The fact that only the Alcalase^®^-treated extract reached the 50% inhibition threshold further underscores the potency of the peptides generated by this enzyme in inhibiting lipase activity. This suggests potential applications of Alcalase^®^ hydrolysates from *P. palmata* as natural anti-obesity agents, which is aligned with growing interest in algal-derived peptides as modulators of metabolic enzymes [15,33]. Conversely, the reduced inhibitory effect seen in Alc+Form may be due to enzyme-enzyme interaction effects, where the presence of Formea^®^ Prime possibly altered the peptide profile, leading to either degradation of key inhibitory peptides or production of less active fragments. Such antagonistic effects in combined enzymatic treatments have been documented previously and emphasize the importance of optimizing enzyme combinations for targeted bioactivity [19,34]. The lack of significant lipase inhibition in extracts treated only with Formea^®^ Prime or without enzymes indicates that trypsin’s narrower specificity and the absence of proteolysis, respectively, are insufficient to produce peptides with lipase inhibitory activity. Notably, the lower inhibition by Formea^®^ Prime compared even to No Enz at certain concentrations could suggest that the peptides generated may interfere with the assay or lack relevant bioactivity, highlighting the complex relationship between peptide structure and enzyme inhibition [1]. Overall, these findings illustrate the critical impact of enzymatic selection on generating functionally active seaweed extracts and suggest Alcalase^®^ as a promising tool for producing seaweed-derived anti-obesity compounds.

The inhibitory activity of the *P. palmata* extracts against α-amylase highlights the role of enzymatic treatment in generating compounds with potential antidiabetic effects. The superior performance of the Alcalase^®^-treated extract suggests that this enzyme facilitates the release of bioactive peptides capable of interacting with the α-amylase active site. This observation aligns with prior studies showing that Alcalase^®^ hydrolysates from algal sources can inhibit carbohydrate-hydrolyzing enzymes, thus supporting their relevance in glycemic control [35,36,37]. A possible explanation for this enhanced activity is the ability of Alcalase^®^ to produce peptides with smaller molecular sizes and a narrower size distribution, which may allow for more effective binding to the α-amylase active site [30]. This interpretation is further supported by the observed DH values (Figure 1), reinforcing the link between peptide size and bioactivity. The diminished inhibition observed in Alc+Form suggests that the addition of Formea^®^ Prime may have altered the peptide profile generated by Alcalase^®^, possibly degrading or diluting key bioactive fractions. Rather than enhancing activity, this enzymatic combination may have led to the formation of less active or non-specific peptides. Similarly, the trypsin-only (Form) treatment showed the lowest inhibitory activity across all concentrations. This result is likely due to Formea^®^ Prime’s narrower substrate specificity and reduced efficiency in releasing relevant bioactive peptides from the seaweed matrix, consistent with previous findings comparing the activities of Alcalase^®^ and trypsin in a seaweed matrix [38]. Interestingly, the non-enzymatic extract retained some α-amylase inhibition, likely due to native compounds present in *P. palmata*, such as polyphenols, known to exhibit mild enzyme inhibitory properties [39]. However, the absence of enzymatic hydrolysis limited the release of additional bioactive peptides, explaining its overall weaker effect compared to Alcalase^®^-treated extracts. Overall, these findings emphasize the importance of enzymatic specificity in designing seaweed hydrolysates with targeted bioactivities. Alcalase^®^ appears particularly effective in generating peptides with α-amylase inhibition activity, offering a potential approach for developing functional ingredients aimed at managing postprandial hyperglycemia.

### 3.3. Inhibitory Effects of Alcalase^®^-Derived Extract Fractions on Metabolic Enzymes

The present study demonstrates that peptide fractions with lower molecular weights (<1 kDa and 1–3 kDa), derived from *P. palmata* extract by using Alcalase^®^, exhibit significantly higher inhibitory activity against porcine pancreatic lipase compared to larger peptides (*p* < 0.05). However, these low-molecular-weight fractions did not exceed the activity of the unfractionated Alcalase^®^ extract, particularly at lower concentrations, where their activity was reduced. This suggests that components contributing to lipase inhibition in the crude extract may be lost during fractionation. One possible explanation is the presence of polyphenol-protein complexes or other non-peptide bioactives [15,40] that remain associated with higher molecular weight peptides in the whole extract and are removed during ultrafiltration. Despite this, the overall trend of enhanced bioactivity in lower molecular weight peptide fractions align with previous studies demonstrating that smaller peptides frequently exhibit stronger pancreatic lipase inhibitory activity [41]. One possible explanation for the superior efficacy of smaller peptides lies in their physicochemical properties. Due to their lower molecular mass and relatively simple structure, short-chain peptides can diffuse more easily through biological membranes and interact more readily with the active or allosteric sites of target enzymes such as pancreatic lipase [42]. Additionally, these peptides may exhibit increased flexibility, allowing for optimal binding conformations that interfere with substrate access or catalytic function [43]. In contrast, the 3–5 kDa and >5 kDa fractions failed to reach 50% inhibition at the maximum concentration tested, suggesting limited interaction or steric hindrance due to their bulkier structure. This outcome reinforces the importance of enzymatic hydrolysis and molecular size fractionation in the production of functionally active peptide mixtures. Furthermore, the limited lipase inhibitory activity of the larger peptide fractions may be attributed not only to the presence of less bioactive or structurally unsuitable sequences for enzyme binding, but also to other factors such as amino acid composition, sequence specificity, and isoelectric point [44]. From a physiological perspective, pancreatic lipase plays a central role in the digestion of dietary triglycerides by catalyzing their hydrolysis into free fatty acids and monoglycerides, which are readily absorbed in the intestine. Therefore, inhibiting this enzyme has become a well-established strategy in obesity management, as it effectively reduces fat absorption and energy intake [1]. Natural lipase inhibitors from dietary sources such as red seaweed offer a promising alternative to synthetic compounds like orlistat, which are often associated with gastrointestinal side effects [45], but further research is needed to investigate whether seaweed derived inhibitors have similar side-effects.

A molecular weight-dependent pattern was also evident in the inhibition of α-amylase, further emphasizing the functional advantages of low molecular weight peptides. The <1 kDa and 1–3 kDa fractions exhibited significantly greater inhibitory activity than the larger peptide fractions (*p* < 0.05), and notably, also outperformed the unfractionated Alcalase^®^ extract. This contrasts with the lipase inhibition results, where the unfractionated extract showed superior activity at lower concentrations. This difference suggests that α-amylase inhibition is more dependent on specific low molecular weight peptides, while lipase inhibition may involve additional components such as polyphenol-protein complexes that are lost during fractionation. The enhanced inhibitory activity may be attributed to the ability of low molecular weight peptides to access and interact more effectively with the enzyme’s active site, likely facilitated by specific amino acid residues that promote stronger binding and interference with the enzyme’s catalytic function [46,47,48]. The lack of 50% inhibition by the 3–5 kDa and >5 kDa fractions at the highest concentration tested further supports the notion that peptide size is a critical determinant of inhibitory potential. Interestingly, the disparity in inhibitory performance between the enzyme systems, particularly the much lower α-amylase inhibition by the >5 kDa fraction compared to its moderate lipase inhibition, suggests that molecular size alone may not fully explain the bioactivity. It is likely that other structural factors such as amino acid composition, sequence motifs, hydrophobicity, and charge distribution also contribute to the peptide-enzyme interactions in a target-specific manner [44]. For example, peptides rich in aromatic or hydrophobic residues have been reported to enhance α-amylase inhibition, possibly through hydrophobic interactions within the enzyme’s active pocket [49]. These findings highlight the potential of low molecular weight peptides from *P. palmata* as dual enzyme inhibitors, targeting both lipase and α-amylase, which are key enzymes in fat and carbohydrate digestion, respectively [1]. Such multifunctional bioactivity could be advantageous for developing functional ingredients aimed at managing obesity and related metabolic disorders. 

### 3.4. In Silico Analysis and Molecular Docking

The combined results of bioactivity prediction, safety screening, motif analysis, and molecular docking led to the identification of four promising peptide candidates with potential inhibitory effects on key digestive enzymes. Specifically, two peptides, SWDGPALVVFT and LDLWKDITF, were predicted to inhibit pancreatic lipase, while ESFNIPAFY and NFYGGKLNGKV were identified as potential inhibitors of pancreatic α-amylase. These peptides originate from a diverse set of proteins in *P. palmata*: SWDGPALVVFT from ferredoxin-dependent glutamate synthase (A0A1C9CH79), LDLWKDITF from Ribulose bisphosphate carboxylase large chain (A0A5Q3RCF2), ESFNIPAFY from Actin (Q8GU34), and NFYGGKLNGKV is from Photosystem II CP47 reaction center protein (A0A1C9CHG0). This diversity demonstrates that bioactive peptides in *P. palmata* can be derived not only from highly abundant photosynthetic proteins, which are likely to contribute substantially to the peptide pool, but also from less abundant metabolic and structural proteins [50]. These findings highlight the broad range of protein sources contributing to potential bioactive sequences and support future efforts to isolate and validate peptides from multiple protein types. All selected peptides demonstrated favorable in silico bioactivity profiles and were non-toxic and non-allergenic according to multiple prediction platforms. These findings suggest that these peptides may serve as viable candidates for the development of functional food ingredients targeting obesity and type 2 diabetes [8]. The physicochemical properties of the peptides (Table 4) offer further insight into their potential bioactivity and bioavailability. All peptides were within the typical molecular weight range for bioactive peptides (9–11 amino acids; 1087–1196 Da), which supports their ability to remain intact during digestion and interact efficiently with enzymatic targets [51]. Interestingly, despite their similar lengths, variations in predicted solubility and net charge were observed. Peptides LDLWKDITF and NFYGGKLNGKV demonstrated favorable predicted solubility, which may enhance their dispersion in aqueous environments and improve access to the enzyme active sites under physiological conditions. Conversely, SWDGPALVVFT and ESFNIPAFY exhibited poor predicted solubility, which may limit their functional efficacy unless encapsulation or delivery strategies are employed [52]. At pH 7, NFYGGKLNGKV was the only peptide with a net positive charge (+2), potentially enhancing electrostatic interactions with the negatively charged surface of α-amylase; its higher isoelectric point (pI = 10.18) may further support favorable interactions under neutral to slightly acidic conditions [53]. In addition, differences in extinction coefficients suggest that lipase-inhibitory peptides, particularly SWDGPALVVFT and LDLWKDITF, may be more readily monitored via UV-absorbance [54,55], which could facilitate their tracking in spectrophotometric or analytical workflows.

Molecular docking provided structural insights into how these peptides might interact with their enzymatic targets. Both SWDGPALVVFT and LDLWKDITF were found to bind effectively near or within the catalytic triad of pancreatic lipase, with LDLWKDITF forming a greater number of hydrogen bonds and hydrophobic contacts. This suggests a more stable and specific interaction with the active site, potentially contributing to stronger inhibition. These interactions are consistent with the peptide’s favorable physicochemical characteristics and high PeptideRanker score [56]. The α-amylase-inhibitory peptides also demonstrated potential binding interactions. ESFNIPAFY and NFYGGKLNGKV might have engaged residues critical for catalysis (Asp197and Glu233) and stabilization (Asp300), which are known to play essential roles in substrate binding and catalytic activity [57]. Notably, NFYGGKLNGKV, despite a lower predicted bioactivity score, showed stable orientation within the binding pocket and strong electrostatic and hydrophobic interactions. This highlights the value of integrating motif-based analysis with docking data, as it can uncover potentially active sequences that may be overlooked by general predictive algorithms [58]. We note that porcine enzymes were used in the *in vitro* assays, whereas human enzymes were employed for docking simulations. While some differences exist between pigs and humans in digestive physiology, including enzyme profiles and intestinal metabolism, the overall structural and functional similarities, as well as comparable patterns of mucosal maturation and tissue architecture, support the use of porcine enzymes as reasonable models for preliminary inhibitory studies [59]. This approach allows us to approximate potential inhibitory activity in humans, though the exact mechanisms underlying these interactions require further experimental validation. Collectively, the docking outcomes support the functional predictions and suggest a structure-activity relationship in which specific residues (e.g., tryptophan, lysine, phenylalanine) and peptide flexibility contribute to effective enzyme inhibition. These findings provide a strong rationale for further *in vitro* validation and could serve as the basis for rational peptide design or optimization to enhance activity, stability, and delivery. It should be noted that the present study did not include enzyme kinetic analyses, which would be necessary to fully elucidate the inhibition mechanism (e.g., competitive or non-competitive). Moreover, while the <3 kDa fractions were enriched in peptides, other constituents such as polyphenols may also contribute to the observed activity. Future studies should therefore incorporate kinetic analyses and controlled experiments to disentangle peptide-specific effects from those of other bioactive components. While *in silico* tools offer a powerful platform for preliminary screening, experimental confirmation remains essential. Future work should therefore include chemical synthesis of the most promising peptides, followed by *in vitro* enzymatic assays to verify inhibitory potency and gastrointestinal stability testing. In parallel, structure-based optimization could be pursued to enhance the inhibitory efficacy of the most promising candidates. Moreover, although our findings demonstrate promising *in vitro* enzyme inhibitory activity of <3 kDa peptide fractions and identify potential bioactive candidates through computational screening, the absence of cellular or animal validation represents a limitation. Future studies should therefore focus on evaluating these peptides in relevant *in vitro* and *in vivo* models to confirm their physiological relevance and therapeutic potential.

## 4. Materials and Methods

### 4.1. Seaweed Biomass Preparation

Air-dried *P. palmata* from a batch collected between late autumn and early winter in 2023 from Faroe Islands coasts was bought from a Danish company (Dansk Tang, Nykøbing Sj., Denmark). The biomass was freeze-dried using a ScanVac CoolSafe freeze-dryer (LaboGene A/S, Allerød, Denmark) and pulverized to approximately 0.5–1.0 cm particle size using a laboratory mill (KN 295 Knifetec™, Foss A/S, Hillerød, Denmark). The resulting powder was stored in zip-lock plastic bags at −20 °C in dark conditions.

### 4.2. Chemicals and Enzymes

Alcalase^®^ (2.4 amino acid units, AU-A, g^−1^) and Formea^®^ Prime (140 kilo mannitol units per gram, KMTU, g^−1^) were kindly provided by Novenesis A/S (formerly known as Novozymes A/S, Bagsværd, Denmark). All solvents used were of high-performance, liquid chromatography (HPLC) grade and purchased from Lab-Scan (Dublin, Ireland). HPLC-grade water was prepared at DTU Food using a Milli-Q^®^ Advantage A10 water deionizing system from Millipore Corporation (Billerica, MA, USA). Porcine pancreatic α-amylase, Acarbose^®^, porcine pancreatic lipase, and Orlistat^®^ were obtained from Sigma–Aldrich (Steinheim, Germany). All other chemicals were obtained from Merck (Darmstadt, Germany).

### 4.3. Extraction Procedure

The enzymatic and alkaline extraction from *Palmaria palmata* was conducted following the procedure used in a previous study [60]. Eight blue-capped bottles (in duplicate) were each loaded with 5 g of biomass powder and 100 mL of deionized water (1:20 *w*/*v*) and incubated in a water bath at 50 °C for 1 h to facilitate biomass rehydration. Post-rehydration, the pH was adjusted to 8 using either HCl (1 M) or Na_2_CO_3_ (1 M). Enzymatic hydrolysis was conducted at 50 °C for 14 h under the following treatment conditions:

- Control (no enzyme)
- Alcalase^®^ at 5% (*w*/*w*, based on biomass protein content)
- Formea^®^ Prime at 5%
- Combined Alcalase^®^ and Formea^®^ Prime (2.5% each)

Following enzymatic incubation, the suspensions were sieved (1 mm mesh), and the filtrates collected and stored at 4 °C. The residual solids were subjected to sequential alkaline extraction (3 cycles) using 80 mL of NaOH/N-acetyl-L-cysteine (NAC) solution (4 g.L^−1^ NaOH, 1 g.L^−1^ NAC), each at room temperature, 130 rpm, for 1.5 h. Solids from each round were re-suspended in fresh alkaline solution, and the resulting supernatants from all three extractions were pooled with the corresponding enzymatic extract. The pH of pooled extracts was adjusted to 8.5–9 to maximize solubility of proteins, peptides, and free amino acids. Samples were pre-frozen at −20 °C for 2 h, followed by −80 °C for 24 h, then freeze-dried (LaboGene A/S, Allerød, Denmark). Final dried extracts were stored at −80 °C in sealed zip-lock bags pending analysis. 

### 4.4. Protein Content Determination

To measure the protein content of samples, the total nitrogen content of the samples was determined via the Dumas combustion method using a fully automated rapid MAX N (Elementar Analysensysteme GmbH, Langenselbold, Germany). About 200 mg of samples were fed into the system, and the exact weight was recorded. The protein content was measured by multiplying the nitrogen content by a factor of 5.0 [60].

### 4.5. Degree of Hydrolysis (DH)

The OPA assay was employed to determine the DH, following the procedure outlined in [61]. OPA reagent was prepared by blending 10 mL of 0.15 M Na_2_CO_3_•10H_2_O with 10 mL of 0.6 M sodium bicarbonate (NaHCO_3_) and 88 mg of dithiothreitol (DTT). Separately, 80 mg of OPA was dissolved in 2 mL of 96% ethanol and subsequently mixed with 10 mL of 1% sodium dodecyl sulfate (SDS). The resulting mixture was combined with the DTT solution and brought to a final volume of 100 mL using distilled water. Samples were diluted to obtain a final protein concentration between 0.05% and 0.25%, then mixed with the OPA reagent in a microplate. Absorbance was recorded at 340 nm, using a calibration curve prepared with L-serine. The serine equivalent of the samples were calculated as outlined below.

$$Sample\left(mg\;Ser.{mL}^{-1}\right)=\frac{\left({Abs}_{sample}-{Abs}_{blank}\right)-intercept}{slope}\times DF$$

*Abs_sample_*, *Abs_blank_*, and DF stand for the sample’s absorbance, the blank’s absorbance, and the dilution factor, respectively. *Intercept* and *slope* are obtained based on the L-serine calibration curve. DH (%) is measured as shown below.

$$DH\left(\%\right)=\frac{Sample\;(\mathrm{mg}\;\mathrm{Ser}.{\mathrm{mL}}^{-1})}{P\times 10}\times 100$$

where *P* represents the protein content in percentage. Each dilution was measured in duplicate.

### 4.6. Porcine Pancreatic Lipase Inhibition Activity

The inhibitory effect of samples on porcine pancreatic lipase was evaluated following the method described in [62] with major modifications as explained in our previous study [15]. A 50 mM solution of p-nitrophenyl butyrate (pNPB) was initially prepared in dimethyl sulfoxide (DMSO) and subsequently diluted with a 0.1 M phosphate buffer (pH 8) to achieve a final concentration of 5 mM. To maintain consistency, all reagents, including the enzyme and test samples, were prepared in the same phosphate buffer. For the positive control, orlistat stock solution was prepared at 1 mg/mL in DMSO and diluted with phosphate buffer (IC_50_ = 0.917 ± 0.012 µg.mL^−1^). In the assay, 40 µL of the sample solution was mixed with 40 µL of porcine pancreatic lipase (0.5 mg.mL^−1^ in phosphate buffer) and incubated at 22 °C for 20 min. Subsequently, 20 µL of the 5 mM pNPB substrate was added, and the reaction mixture was incubated at 37 °C for an additional 30 min. The absorbance was then measured at 410 nm using an Eon^TM^ microplate spectrophotometer (BioTek Instruments, Inc., Winooski, VT, USA). The inhibition percentage of pancreatic lipase by samples was calculated as follows:

$$Pancreatic\;lipase\;inhibition\;(\%)=\left(1-\frac{A_{s}}{A_{b}}\right)\times 100$$

where *A_s_* and *A_b_* stand for absorbance of sample and blank, respectively.

### 4.7. Porcine Pancreatic α-Amylase Inhibition Activity

The inhibitory activity of samples against porcine pancreatic α-amylase was evaluated according to the method outlined in [63]. Briefly, 30 µL of each sample, prepared in 30 mM Sorensen’s phosphate buffer (pH 7), was mixed with 30 µL of α-amylase solution (0.5 mg.mL^−1^) in the same buffer and incubated at 37 °C for 20 min. A 0.5% (*w*/*v*) starch (Sigma-Aldrich, St. Louis, MO, USA) solution was prepared by dissolving starch in 30 mM Sorensen’s phosphate buffer (pH 7), followed by boiling with stirring for 10 min. After the initial enzyme incubation, 60 µL of the starch solution was added to each well, and the reaction mixtures were incubated at 37 °C for an additional 10 min. The reaction was terminated by the addition of 120 µL of dinitrosalicylic acid (DNS) reagent. The DNS reagent was prepared by dissolving 0.25 g of DNS in 5 mL of 2 N NaOH, then diluting with deionized water to 12 mL. Subsequently, 7.5 g of sodium tartrate was added, and the final volume was brought up to 25 mL. The 96-well plate containing the reaction mixtures was incubated at 100 °C for 7 min, followed by cooling to room temperature. Absorbance was measured at 540 nm. Acarbose served as the positive control (IC_50_ = 8.996 ± 0.030 µg.mL^−1^). The inhibition percentage of pancreatic α-amylase by samples was calculated as follows:

$$Pancreatic\;\alpha -amylase\;inhibition\;(\%)=\left(1-\frac{A_{s}}{A_{b}}\right)\times 100$$

where *A_s_* and *A_b_* denote absorbance of sample and blank, respectively.

### 4.8. Fractionation of Extracts Through Ultrafiltration

The chosen extract solution was fractionated based on molecular weight using a 300 mL stirred ultrafiltration cell (Millipore, NH, USA), equipped with 76 mm Ultracel^®^ regenerated cellulose membranes with nominal molecular weight cut-offs (MWCO) of 5 kDa, 3 kDa, and 1 kDa (Millipore, Jaffrey, NH, USA). Fractionation was carried out in a stepwise manner. Initially, the extract was subjected to ultrafiltration through the 5 kDa membrane under a constant nitrogen pressure of 5 bar. The resulting permeate was subsequently passed through 3 kDa and then 1 kDa membranes to further separate lower molecular weight components. This sequential filtration yielded four distinct peptide fractions: <1 kDa, 1–3 kDa, and 3–5 kDa, and >5 kDa. The resulting fractions were freeze-dried and stored at −80 °C until analyses.

### 4.9. Peptide Purification Through Size Exclusion Chromatography (SEC)

After ultrafiltration, SEC was employed to increase peptide purity. A HiPrep 16/60 Sephacryl S-500 HR column (GE Healthcare, Chicago, IL, USA) was used with PBS buffer (1 mL/min) on an ÄKTA Pure system. To this end, the samples were centrifuged, and 1 mL of the resulting supernatant was injected onto the column. The main peak of the chromatogram, corresponding to the peptide-containing fraction, was collected to separate it from potential higher molecular weight contaminants such as large proteins or protein aggregates. This step ensured a higher degree of purity and homogeneity in the preparation for downstream applications. It should be noted that for the inhibition assays, the ultrafiltrated extracts were used rather than the SEC-purified peptides.

### 4.10. Peptide Identification Using Liquid Chromatography-Tandem Mass Spectrometry (LC-MS/MS)

Peptide samples were acidified to 1% trifluoroacetic acid (TFA) and desalted using SOLAµ SPE plates (HRP, Thermo Fisher Scientific, Waltham, MA, USA) with solvent exchanges performed via centrifugation. The filters were activated with methanol and acetonitrile/formic acid, equilibrated with 1% TFA/3% acetonitrile, and then loaded with samples. Peptides were washed, eluted with 40% acetonitrile/0.1% formic acid, dried in a SpeedVac, and reconstituted in 12 µL of Solution A* (2% acetonitrile, 1% TFA). After centrifugation (18,000× *g*, 10 min), peptide concentration was determined by Nanodrop (Thermo Fisher Scientific, Waltham, MA, USA). Peptides were loaded onto a 2 cm C18 trap column (Thermo Fisher Scientific, Waltham, MA, USA; Cat. No. 164946), connected in-line to a 15 cm C18 reverse-phase analytical column (Thermo EasySpray ES904, Thermo Fisher Scientific, Waltham, MA, USA) using 100% Buffer A (0.1% formic acid in water) at 750 bar, with the column oven operating at 30 °C on a Thermo EasyLC 1200 HPLC system (Thermo Fisher Scientific, Waltham, MA, USA). Peptides were eluted over a 70 min gradient ranging from 6 to 60% Buffer B (80% acetonitrile, 0.1% formic acid) at 250 nL.min^−1^. Data were acquired on a Q-Exactive mass spectrometer (Thermo Fisher Scientific, Waltham, MA, USA) in data-dependent MS2 (Top10) mode. MS1 spectra were recorded at 70,000 resolution, and MS2 at 17,500, with dynamic exclusion (30 s) and exclusion of charge states < 2. Raw files were analyzed using Proteome Discoverer version 2.4 (Thermo Fisher Scientific, Waltham, MA, USA), with dynamic modifications set for oxidation (M), acetylation (K), N-terminal acetylation, and Met-loss. SequestHT (Thermo Fisher Scientific, Waltham, MA, USA) was used for database searching against the UniProt reference proteome plus recombinant sequences, with a delta Cn filter of 0.05 for peptide-spectrum matches (PSMs).

### 4.11. In Silico Analysis of Purified Peptides

#### 4.11.1. Peptide Prediction of Bioactivity

Identified peptides were screened for their potential bioactivity using PeptideRanker (http://distilldeep.ucd.ie/PeptideRanker/) (accessed on 27 June 2025), a neural network-based tool that scores peptides on a scale from 0 to 1, with higher values indicating greater probability of bioactivity. Peptides scoring above 0.6 were shortlisted for further analysis.

#### 4.11.2. Toxicity Prediction

The shortlisted peptides were subjected to toxicity assessment using ToxinPred (https://webs.iiitd.edu.in/raghava/toxinpred/protein.php) (accessed on 28 June 2025). This tool utilizes machine learning models trained on known toxic and non-toxic peptides to predict potential toxicity. Peptides predicted to be non-toxic were retained for further allergenicity screening.

#### 4.11.3. Allergenicity Assessment

Allergenicity of the candidate peptides was evaluated using two complementary tools: AllerCatPro 2.0 (https://allercatpro.bii.a-star.edu.sg/) (accessed on 28 June 2025), a structure- and sequence-based allergenicity predictor that uses 3D similarity and known allergen motifs; and AllergyPred (https://allergypred.charite.de/AllergyPred/) (accessed on 28 June 2025), a support vector machine-based method for predicting peptide allergenicity from sequence. Peptides classified as non-allergenic by both tools were considered safe for further application.

#### 4.11.4. Anti-Obesity and Antidiabetic Activity Prediction

To identify potential enzyme inhibitors, the validated peptides were screened using BIOPEP-UWM (https://biochemia.uwm.edu.pl/en/biopep-uwm-2/) (accessed on 14 July 2025), a comprehensive database of bioactive peptide sequences and their associated activities. The peptides were analyzed for known motifs linked to pancreatic α-amylase and pancreatic lipase inhibition. Those with the highest matching scores and known inhibitory activities were selected for *in silico* docking analysis.

#### 4.11.5. Molecular Docking

To evaluate the interaction of selected peptides with their target enzymes, molecular docking was performed using ClusPro 2.0 (https://cluspro.org/) (accessed on 17 September 2025) and SwissDock (http://www.swissdock.ch/) (accessed on 10 August 2025), online docking platforms. The 3D structures of the selected peptides were generated using PEP-FOLD3 (https://bioserv.rpbs.univ-paris-diderot.fr/index.html) (accessed on 10 August 2025) and converted to the appropriate format for docking (MOL2 or PDB).

The crystal structures of human pancreatic α-amylase (PDB ID: 1HNY) and pancreatic lipase (PDB ID: 2PPL) were retrieved from the Protein Data Bank (https://www.rcsb.org/) (accessed on 10 August 2025). It is noteworthy that porcine α-amylase and lipase were used in the *in vitro* inhibition assays due to their commercial availability and established use in preliminary enzymatic studies. For molecular docking, human enzyme structures were employed because high-resolution crystal structures are available, allowing for accurate computational modeling. Porcine and human enzymes share high sequence and structural homology, particularly in the active sites, making porcine enzymes reasonable proxies for human enzymes in preliminary inhibitory studies. Each peptide was docked separately to the respective enzyme. The resulting docking poses were ranked based on binding energy, interaction stability, and binding site relevance.

From the docking results, the two peptides with the lowest (most favorable) binding energy and predicted inhibitory activity against each target enzyme were reported as the most promising bioactive candidates and were visualized using Discovery Studio Visualizer Software (BIOVIA, Dassault Systèmes, San Diego, CA, USA).

### 4.12. Statistical Analysis

The data were analyzed using Analysis of Variance (ANOVA) for comparisons involving more than two groups, followed by Tukey’s post hoc test to determine differences between means. For comparisons involving only two groups, a two-sample t-test was performed. All statistical analyses were conducted using OriginPro 2023 (OriginLab Co., Northampton, MA, USA). Differences were considered statistically significant at *p* < 0.05.

## 5. Conclusions

The results demonstrate that Alcalase^®^-mediated hydrolysis is an effective strategy for generating bioactive peptides from *P. palmata*, capable of inhibiting lipase and α-amylase activities, which are key targets in obesity and type 2 diabetes management. Low-molecular-weight peptides (<3 kDa) were particularly potent, and bioinformatic analyses confirmed their safety and predicted bioactivity. Notably, 51 peptides scored above 0.6 on PeptideRanker, with selected sequences showing strong docking affinity to metabolic enzymes. Despite these promising findings, this study is limited to *in vitro* enzyme inhibition and *in silico* predictions. Future research should include *in vivo* validation, stability assessments under gastrointestinal conditions, and peptide isolation and synthesis for structure-activity relationship studies. Additionally, the interaction of these peptides within complex food matrices remains unexplored. Nonetheless, this work provides a strong foundation for the development of marine-derived functional food ingredients or nutraceuticals, particularly from red seaweed, aimed at supporting metabolic health.

## Figures and Tables

**Table 1:** Percentage inhibition of porcine pancreatic lipase by *P. palmata* extracts obtained via sequential enzymatic/alkaline treatments.

Extract (mg.mL^−1^)	No Enz	Alc	Form	Alc+Form
4	16.74 ± 1.01 ^b,v^	51.20 ± 0.33 ^a,v^	22.23 ± 2.89 ^b,v^	46.72 ± 4.21 ^a,v^
2	15.54 ± 0.50 ^c,v^	50.23 ± 0.62 ^a,vw^	16.84 ± 0.69 ^c,w^	29.00 ± 3.12 ^b,w^
1	14.44 ± 1.87 ^c,v^	47.62 ± 2.59 ^a,vw^	11.89 ± 1.43 ^c,x^	22.55 ± 2.59 ^b,wx^
0.5	8.69 ± 1.50 ^c,w^	43.19 ± 1.25 ^a,w^	4.18 ± 2.19 ^d,y^	19.60 ± 0.59 ^b,xy^
0.25	7.66 ± 2.38 ^c,w^	33.88 ± 2.52 ^a,x^	2.57 ± 1.30 ^c,y^	14.11 ± 1.23 ^b,y^
0.125	1.41 ± 0.93 ^b,x^	19.19 ± 5.21 ^a,y^	1.78 ± 0.87 ^b,y^	6.59 ± 1.35 ^b,z^

Data were expressed as mean ± standard deviation (*n* = 3). The superscripts a–d indicate significant differences among the treatments at each concentration tested, and the superscripts v–z indicate significant differences among the concentrations for each treatment (*p* < 0.05). No Enz, Alc, Form, and Alc+Form refer to treatments with no enzyme, Alcalase^®^, Formea^®^ Prime, and a combination of Alcalase^®^ and Formea^®^ Prime, respectively.

**Table 2:** Percentage inhibition of porcine pancreatic α-amylase by *P. palmata* extracts obtained via sequential enzymatic/alkaline treatments.

Extract (mg.mL^−1^)	No Enz	Alc	Form	Alc+Form
4	21.97 ± 1.70 ^b,x^	45.00 ± 4.52 ^a,x^	3.71 ± 1.67 ^c,x^	23.44 ± 1.42 ^b,x^
2	15.94 ± 2.07 ^b,y^	36.39 ± 5.96 ^a,x^	4.09 ± 0.26 ^c,x^	13.16 ± 2.91 ^bc,y^
1	12.22 ± 0.23 ^a,y^	13.69 ± 1.68 ^a,y^	1.05 ± 0.37 ^c,y^	7.19 ± 2.12 ^b,z^

Data were expressed as mean ± standard deviation (*n* = 3). The superscripts a–c indicate significant differences among the treatments at each concentration tested, and the superscripts x–z indicate significant differences among the concentrations for each treatment (*p* < 0.05). No Enz, Alc, Form, and Alc+Form refer to treatments with no enzyme, Alcalase^®^, Formea^®^ Prime, and a combination of Alcalase^®^ and Formea^®^ Prime, respectively.

**Table 3:** Percentage inhibition and IC_50_ (mg.mL^−1^) of molecular weight-based fractions of *P. palmata* enzymatic/alkaline extract obtained using Alcalase^®^ against porcine pancreatic lipase and α-amylase.

		<1 kDa	1–3 kDa	3–5 kDa	>5 kDa
**Lipase** **Inhibition**	4 mg.mL^−1^	54.59 ± 0.87 ^a,x^	53.26 ± 0.51 ^a,x^	46.27 ± 0.65 ^b,x^	41.33 ± 0.78 ^c,x^
	2 mg.mL^−1^	43.07 ± 2.01 ^a,y^	41.12 ± 0.81 ^a,y^	32.01 ± 1.39 ^b,y^	30.49 ± 0.73 ^b,y^
	1 mg.mL^−1^	33.51 ± 2.06 ^a,z^	30.89 ± 2.02 ^a,z^	20.57 ± 3.11 ^b,z^	14.80 ± 1.48 ^b,z^
	IC_50_	3.25 ± 0.04 ^a^	3.47 ± 0.05 ^b^	NR *	NR
**α-amylase Inhibition**	4 mg.mL^−1^	58.25 ± 0.95 ^a,x^	55.11 ± 0.79 ^a,x^	29.68 ± 3.91 ^b,x^	6.78 ± 1.07 ^c,x^
	2 mg.mL^−1^	36.12 ± 1.70 ^a,y^	32.65 ± 2.70 ^a,y^	16.27 ± 2.27 ^b,y^	5.41 ± 0.59 ^c,x^
	1 mg.mL^−1^	28.15 ± 2.46 ^a,z^	22.47 ± 2.11 ^b,z^	7.16 ± 1.43 ^c,z^	1.39 ± 0.74 ^d,y^
	IC_50_	3.24 ± 0.09 ^a^	3.55 ± 0.07 ^b^	NR	NR

Data were expressed as mean ± standard deviation (*n* = 3). The superscripts a–d indicate significant differences among the treatments at each concentration tested, and the superscripts x–z indicate significant differences among the concentrations for each treatment (*p* < 0.05). Additionally, superscripts a,b in the IC_50_ rows indicate significant differences between the <1 kDa and 1–3 kDa fractions in terms of lipase and α-amylase inhibitory activity (*p* < 0.05). * Not Reached (IC_50_ could not be calculated because 50% inhibition was not achieved at the highest concentration tested).

**Table 4:** Physicochemical properties of selected bioactive peptides from sequential Alcalase^®^/alkaline treated *P. palmata* targeting digestive enzymes.

Sequence	Target	Number of Residues	Molecular Weight (g.mol^−1^)	Extinction Coefficient (M^−1^cm^−1^)	Isoelectric Point	Net Charge at pH 7	Solubility (Estimation)
SWDGPALVVFT	Lipase inhibitor	11	1191.33	5690	0.75	−1	Poor water solubility
LDLWKDITF	Lipase inhibitor	9	1150.32	5690	3.71	−1	Good water solubility
NFYGGKLNGKV	α-amylase inhibitor	11	1196.35	1280	10.18	2	Good water solubility
ESFNIPAFY	α-amylase inhibitor	9	1087.18	1280	0.95	−1	Poor water solubility

## Data Availability

The data acquired in this study can be obtained at request.

## Supplementary Materials

The following supporting information can be downloaded at: https://www.mdpi.com/article/10.3390/md23100392/s1, Table S1: Predicted bioactive peptides from *Palmaria palmata*, their bioactivity scores, and parent protein accession numbers.
